# Pirfenidone mitigates demyelination and electrophysiological alterations in multiple sclerosis: Targeting NF-κB, sirt1, and neurotrophic genes

**DOI:** 10.1007/s00210-024-03496-8

**Published:** 2024-10-15

**Authors:** Reda A. A. Abo-Elsoud, Eman A. Ali, Marwa A. Al-Gholam, Mohamed S. Rizk, Rasha S. A. Elseadawy, Omnia Ameen

**Affiliations:** 1 Medical Physiology Department, Faculty of Medicine, Menoufia University, Shebin El Kom, Menoufia, Egypt; 2 Clinical Pharmacology Department, Faculty of Medicine, Menoufia University, Shebin El Kom, Menoufia, Egypt; 3 Anatomy and Embryology Department, Faculty of Medicine, Menoufia University, Shebin El Kom, Menoufia, Egypt; 4 Medical Biochemistry and Molecular Biology Department, Faculty of Medicine, Menoufia University, Shebin El Kom, Menoufia, Egypt; 5 Medical Biochemistry and Molecular Biology Department, Faculty of Dentistry, AlRyada University, Sadat City, Menoufia, Egypt; 6 Neuropsychiatry Department, Faculty of Medicine, Menoufia University, Shebin El Kom, Menoufia, Egypt

**Keywords:** Multiple sclerosis, Cuprizone, Pirfenidone, SIRT1, NGF, Neuregulin-1

## Abstract

Multiple sclerosis (MS) is a demyelinating disease affecting the central nervous system associated with progressive neurodegeneration. Pirfenidone (Pir) is a well-known antifibrotic agent; however, Pir’s function in MS is little explored. We evaluated the neuroprotective effects of Pir in MS and its possible underlying mechanisms. Forty male Swiss mice were divided equally into control, cuprizone (CPZ), Pir, and CPZ + Pir groups. Assessment of motor function was conducted using neurobehavioral tests, EMG, and nerve conduction velocity (NCV). Mice’s brains were extracted to measure oxidative stress, neuroinflammatory markers, and the expression of neurotrophic genes. The corpus callosum and the sciatic nerve were subjected to histopathological and immunohistochemical studies. The CPZ group was associated with significant reductions in muscle power, frequency of contraction, sciatic NCV, SOD, IL-10, SIRT1, NGF, and neuregulin-1. Significant increases in MDA, TNF-α, INF-γ, IL-17, TGF-β, and NF-κB were also detected. Multiple areas of partially demyelinated nerve fibers in the corpus callosum, the loss of oligodendrocyte nuclei, and increased microglia and astrocytes were also observed. The sciatic nerve revealed partial demyelination with significantly reduced myelin basic protein (MBP) expression. Pir significantly restored motor function, demyelination, and neurodegenerative changes induced by CPZ. Besides the antifibrotic action of Pir, we concluded that it improves motor function in MS by alleviating the demyelinating process and neurodegeneration. Its potential anti-inflammatory, antioxidant, and antifibrotic properties could be contributing factors. These effects could be mediated by modulating the NF-κB, SIRT1, NGF, and neuregulin-1 pathways. Pir is a promising agent for treating MS.

## Introduction

Multiple sclerosis (MS) is a chronic inflammatory demyelinating disease of the brain and spinal cord. It is characterized by recurrent demyelination and incapacitating effects. It frequently leads to neuronal death and irreversible axonal damage. It is mainly diagnosed in young 20–40-year-old adults. Approximately 2.5 million MS patients exist worldwide, and that figure is steadily rising (Pegoretti et al. 2020). Most MS patients have neurological impairment, resulting in problems with walking, vision, muscle coordination, and other body functions. These symptoms have a significantly negative impact on patients’ and caregivers’ health-related quality of life. MS has no known treatment, and its prognosis is uncertain (Thirion et al. 2023).

Multiple sclerosis has a complicated and poorly known etiology. Demyelinating plaques in the white and grey matter of the central nervous system (CNS) are the primary causes of MS pathology. The demyelinating lesions are thought to be caused by an autoimmune-type inflammatory response. Neurological impairment, indicated by demyelination and axonal conduction block, is associated with the early stage of inflammatory cells and activated microglia infiltrates, followed by an overproduction of inflammatory mediators (Pegoretti et al. 2020). The pathogenic initiators of MS are thought to be interleukin-17 (IL-17) and interferon-gamma (IFN-γ) (Pegoretti et al. 2020).

Furthermore, earlier studies demonstrated that MS pathophysiology is associated with noticeably higher amounts of tumor necrosis factor-alpha (TNF-α), also linked to the disease’s progression (Baecher-Allan et al. 2018). Oxidizing radicals like superoxide, hydroxyl radicals, and hydrogen peroxide can be produced in large quantities by activated microglia and macrophages. Chronic oxidative stress has a significant role in the clinical features of MS, including inflammation, axonal degeneration, and myelin degradation. Oligodendroglia, myelin-forming cells, are susceptible to death due to oxidative stress. Oxidative stress indicators were higher in the cerebrospinal fluid (CSF) of MS patients (Ramos-González et al. 2024).

Recent research has illustrated that antagonistic crosstalk mediates the control of energy metabolism and innate immunity between Sirtuin 1 (SIRT1) and nuclear factor κB (NF-κB) signaling pathways. Apoptosis, differentiation, proliferation, and inflammation have all been linked to the NF-κB signaling cascade. NF-κB is regarded as a vital regulator of the inflammatory response due to its ability to promote the production of proinflammatory cytokines. MS patients have an overactive NF-κB signaling pathway (Safa et al. 2021). SIRT1 controls cellular survival and oxidative respiration. SIRT1 activation inhibits NF-κB signaling, resulting in the resolution of inflammation and promoting oxidative balance (Kauppinen et al. 2013). According to the published research, SIRT1 activation may have neuroprotective effects on various neurodegenerative illnesses, such as Alzheimer’s and Parkinson’s disease (Sharma et al. 2021b). Nerve growth factor (NGF) regulates neuronal survival, differentiation, and neuroinflammation in the brain. NGF is known to enhance oligodendrogenesis and promote myelination (Brandi et al. 2021). Neuregulin-1 is a neurotrophic factor that promotes neuronal survival and supports axonal and neuromuscular development and maintenance (Mòdol-Caballero et al. 2021).

The disabilities linked to MS cannot be adequately stabilized or reversed with the current treatment. Pirfenidone (Pir), a small pyridine molecule, has been licensed to treat idiopathic pulmonary fibrosis due to its notable antifibrotic effect. Pir readily passes across the blood-brain barrier because it is a tiny molecule. Pir’s unique pharmacological profile and mode of action have attracted attention recently, leading to its suggestion for extrapulmonary illnesses (Sartiani et al. 2022). Pir contains antioxidant and anti-inflammatory qualities in addition to its antifibrotic action. It reduces TNF-α production and blocks TNF-α receptors, the main cytokine involved in demyelination (Walker and Margolin 2001). In acute pancreatitis, Pir triggered interleukin-10 (IL-10) secretion (Palathingal Bava et al. 2022).

Moreover, Pir can exert its antioxidant effect by scavenging reactive oxygen species (ROS) and inhibiting lipid peroxidation (Fois et al. 2020). In the therapy of cardiac remodeling, diabetic nephropathy, loss of hippocampal neurons, dementia, and head injuries, pirfenidone has demonstrated encouraging outcomes (Castro-Torres et al. 2014). Another study revealed that Pir’s anti-inflammatory and antioxidant properties mitigate the neuron loss caused by the excitotoxicity of kainic acid in the rat hippocampal tissues (Castro-Torres et al. 2015).

Based on integrating points between Pir’s pharmacological mechanisms and the pathogenesis of MS, the current study aims to investigate Pir’s potential neuroprotective benefits in experimentally-induced MS in mice and the possible molecular mechanisms mediating its action.

## Materials and methods

### Experimental animals

Forty male Swiss mice weighing (20–30 g) were brought from Theodore Bilharz Research Institute (Giza, Egypt) and acclimatized (5 mice/cage), with free access to water and diet, in Menoufia Faculty of Medicine animal house for 7 days before commencing the research. Environmental conditions were set at a 12-h light/dark cycle, temperature of 22±2 °C, and humidity of 40–70%. The work adhered to The Guide of Care and Use of Laboratory Animals and the ARRIVE guidelines and was approved by the Menoufia Faculty of Medicine ethical committee (Registry No. 7/2024 BIO 1).

### Study design

According to the study of Naeem et al., the sample size calculated using G*Power software (Aichach, Germany) was forty mice. The power of the study was 80%, and the confidence level was 95% (Naeem et al. 2022). The mice were allocated at random into the following four groups (10/group):

1. The control (Cont.) group: 1% carboxymethyl cellulose (CMC) was administered to the mice via oral gavage for 5 weeks. 1% CMC was obtained by thoroughly mixing one g of CMC with 100 ml of distilled water at a temperature of 60 °C (Lan et al. 2018). The resulting solution was freshly prepared every week and kept at 4 °C.
2. The pirfenidone (Pir) group: Pir (Pirfenex 200 mg tablets, Cipla Ltd, Mumbai, India) was administered to the mice at a dose of 300 mg/kg/day via oral gavage dissolved in 1% CMC and given as 40 ml/kg for 5 weeks (Liu and Shi 2019).
3. The cuprizone (CPZ) group: CPZ (C9012-25G, Sigma Aldrich, St. Louis, MO, USA) was administered to the mice at a 400 mg/kg/day dose via oral gavage dissolved in 1% CMC to be given as 40 ml/kg for 5 weeks (Zhen et al. 2017).
4. The pirfenidone + cuprizone (Pir + CPZ) group: Pir was administered to the mice half an hour before giving CPZ for 5 weeks by the same doses and routes in Pir and CPZ groups.

**Fig. 1:**
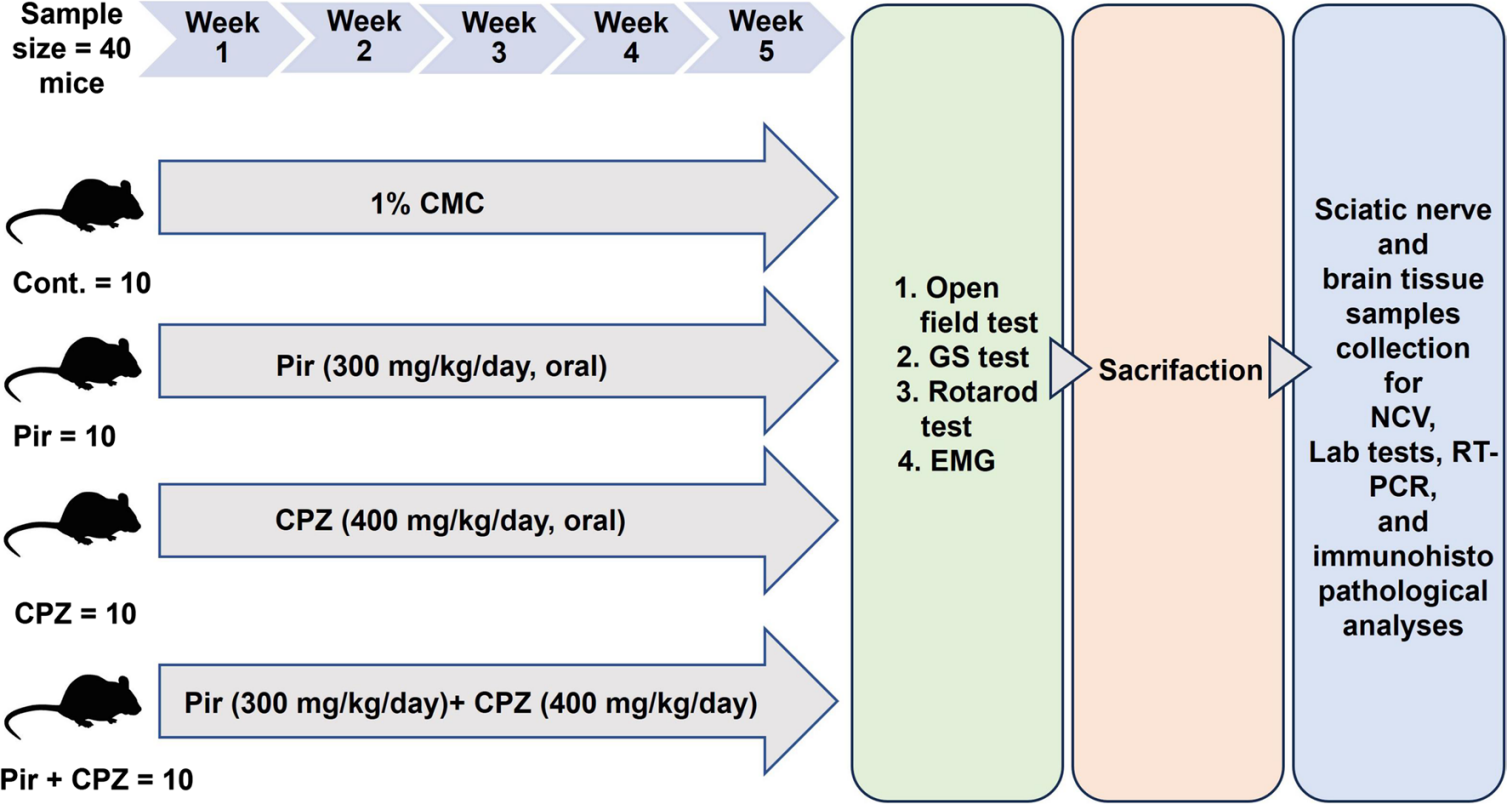
Schematic diagram of the experimental design. Cont., control; CMC, carboxymethylcellulose; CPZ, cuprizone; EMG, electromyography; GS, grip strength; NCV, nerve conduction velocity; Pir, pirfenidone; RT-PCR, real-time polymerase chain reaction

At the end of the experiment, mice were assessed for motor function using neurobehavioral tests (open field, grip strength (GS), and rotarod tests) to determine anxiety, motor coordination, muscular strength, and locomotor activity. In addition, electrophysiological tests using nerve conduction velocity (NCV) and electromyography (EMG) were performed. Mice were then anesthetized and sacrificed by cervical elongation and dislocation. The brain was removed and rinsed with phosphate-buffered saline (PBS) (pH 7.4). The left hemisphere was weighed and separated into two halves. One half was utilized for the biochemical study of inflammatory and oxidative stress markers, while the other half was utilized for real-time polymerase chain reaction (RT-PCR) experiments. The right hemisphere and the sciatic nerve were utilized for histopathological and immunohistochemical examinations. The schematic diagram shows the experimental design (Fig. 1).

### Neurobehavioral tests

#### Open field test

A white wooden arena (45 cm × 60 cm × 50 cm) was designed to perform the open field test. Black lines were drawn to divide the arena’s bottom into nine equal partitions, each measuring 15 cm × 20 cm. Then, mice were cautiously positioned in the left near quadrant and left for 5 min to explore the arena freely. The number of rearing, grooming, center crossing, and crossed squares were executed by each mouse over 5 min. The test chamber was cleaned with 70% ethanol between mice **(**Arent et al. 2015**)**.

#### Rotarod test

Three days before the test day, mice were allowed to train in the rotarod apparatus, with an average of four daily trials. On the test day, mice underwent a three-session test, each lasting 180 s at maximum. Mice were noticed for the number of falls during each test, and the average was calculated to get the number of falls. Moreover, the latency was calculated by dividing the two most prolonged periods each mouse spent on the spinning rod by 180 and then multiplied by 100 to obtain the percentage **(**Deacon 2013**)**.

#### Grip strength (GS) test

The researchers employed a straightforward and noninvasive approach to assess the mice’s GS, a test for rodents’ motor capabilities examination. The experimental setup involved centering each mouse on a slim horizontal steel wire. The wire had a diameter of 5 mm and measured 40 cm in length and height. The duration (latency) for which each mouse maintained its grip on the wire until it lost balance was recorded **(**Takeshita et al. 2017**)**.

### Electrophysiological assessment

#### Electromyography (EMG)

The MP 30 Ultimate System ® (BIOPAC, Aero Camino, Goleta, CA, USA) was utilized to carry out EMG. During the procedure, mice were kept under xylazine and ketamine (10 and 35 mg/kg, respectively) anesthesia. BIOPAC BSL PRO 3.6.7 software was utilized to analyze the obtained numerical data. EMG recordings from the m. gastrocnemii were obtained using needle recording electrodes (BIOPAC EL 254 S and EL 254). The electrodes were strategically placed on the origin and insertion areas of the muscle, with a grounding electrode positioned at the tail root. To elicit a response, a series of five consecutive stimuli, each lasting 0.1 ms, were administered at a frequency of 1.0 stimulus per second. The stimulation intensity was carefully adjusted to achieve the maximum response. The records from each stimulation series were averaged, effectively reducing noise and smoothing the response curves to enhance data quality **(**Ünsal and Özcan 2018**)**.

#### Nerve conduction velocity (NCV)

The right sciatic nerve was dissected from its origin in the spinal cord and down to the knees. Then, it was removed by cutting at its proximal and distal ends. The sciatic nerve was stimulated in a ringer solution pre-filled nerve chamber connected to the Power Lab 4/30 (ADInstruments Pty Ltd, NSW, Australia) recording unit. The analysis was conducted using Lab Chart software (ADInstruments Pty Ltd, NSW, Australia). To compute NCV, the distance (m) between the stimulating and the recording electrodes was estimated and then divided by the time (s) interval between a stimulus artifact and the onset of the response **(**El Agamy et al. 2023**)**.

### Biochemical analysis

Brain tissue was perfused with PBS solution and then homogenized in a 5-ml cold buffer per gram tissue. Centrifugation of the brain tissue was performed at 4000 rpm for 15 min; then, the supernatant was removed and stored at – 80 °C. The colorimetric method was employed to detect the malondialdehyde (MDA) (ab118970) in the brain homogenate provided by Abcam Co., Cambridge, UK. Meanwhile, the superoxide dismutase (SOD) (MBS070857) level was detected using Eliza kits from MyBioSource Inc. (San Diego, CA, USA). INF-γ (ab100690, Abcam Co., Cambridge, UK), TNF-α (MBS825075), IL-10 (MBS704754), IL-17 (MBS2508197), and transforming growth factor-β1 (TGF-β1) (MBS160136) levels in the brain homogenate were detected using ELIZA kits from MyBioSource Inc. (San Diego, CA, USA). All markers were measured according to the manufacturer’s instructions.

### Real-time polymerase chain reaction (RT-PCR)

Total RNA was extracted from the brain tissues using Qiagen RN Easy Plus Universal Kit (USA). After that, RNA was checked to confirm its quality and purity and kept at − 80 °C until used. The PCR technique included two successive procedures. The first procedure was cDNA synthesis by QuantiTect Reverse Transcription Kit (Qiagen, USA) utilizing one cycle of Applied Biosystems 2720 thermal cycler (Singapore). GAPDH was employed as a reference gene. The second procedure included cDNA amplification. cDNA was utilized in SYBR green-based quantitative RT-PCR for relative quantification (RQ) of NF-κB, SIRT1, NGF, and neuregulin-1 genes expression using SensiFASTTMSYBR Lo-ROX Kit (USA). Genes’ primers are provided in Table 1. Eventually, Applied Biosystems 7500 software version 2.0.1 was utilized to analyze data. The RQ of NF-ҝB, SIRT1, NGF, and neuregulin-1gene expression included normalizing the amount of these genes to a reference gene (GAPDH) relative to a control sample using the ∆∆Ct method.

**Table 1:** Primers used for measuring the expression of NF-κB, SIRT1, NGF, and neuregulin-1 genes

Genes	Forward	Reverse
NF-KB	GAAATTCCTGATCCAGACAAAAAC	ATCACTTCAATGGCCTCTGTGTAG
SIRT 1	AGA AACAATTCCTCCACCTGA	GCTTTGGTGGTTCTGAAAGG
NGF	GGCATGCTGGACCCAAGCTC	GCGCTTGCTCCGGTGAGTCC
Neuregulin-1	CAGGAACTCAGCCACAAACA	CCCAGTCGTGGATGTAGATGT
GAPDH	TGCACCACCAACTGCTTAGC	GGCATGGACTGTGGTCATGAG

### Histopathological and immunohistochemical studies

A hematoxylin and eosin stained 5-μm-thick paraffin section was prepared from specimens (brain and sciatic nerve) fixed in 10% formalin solution (Feldman and Wolfe 2014). Embedded paraffin was created from dehydrated brain tissue and sciatic nerves fixed with paraformaldehyde in PBS overnight for immunohistochemistry. In brief, deparaffinized sections were rinsed three times with 0.01 mol/L PBS after preincubation with citrate buffer. H2O2 was applied to sections at a concentration of 0.3%. The sections underwent a 1-h incubation with 3% BSA for 1 h at 37 °C to halt non-specific binding. Subsequently, the sections were exposed to the following primary antibodies from Abcam Co. (Cambridge, UK): anti-myelin basic protein (MBP) (ab40390, Rabbit polyclonal, 1:300, Abcam, UK), anti-oligodendrocyte transcription factor 2 (Olig2) (ab109186, Rabbit monoclonal, 1:100), anti-ionized calcium-binding adapter molecule1 (Iba1), a marker for microglia (ab108539, Rabbit polyclonal, 1:1200), anti-glial fibrillary acidic protein antibody (GFAP), and a marker for astrocyte activation (ab7260, Rabbit polyclonal, 1:300). Succeeding a 10-min incubation with the biotinylated secondary antibody, the sectioned tissues were rinsed with PBS. Streptavidin HRP was utilized as a rinse after three PBS washes. Immunoreactivity was visualized using DAB for 30 min. Subsequently, the slides were counterstained with Mayer’s hematoxylin and mounted with a cover slip. Photomicrographs were captured with an Olympus light microscope **(**Ramos-Vara et al. 2008**)**.

### Morphometric study

Ten non-overlapping fields (x400) of each group were examined at the Anatomy Department, Faculty of Medicine, Menoufia University. The same investigator conducted all measurements to avoid inter-observer errors. Images J 1.47 v software (USA) was utilized for morphometric measurements of the area percentage of immunoexpression of MBP and the number of Olig2, Iba1, and GFAP-positive cells.

### Statistical analysis

After collecting data for analysis, they were found to fulfill the parametric assumptions (according to Shapiro-Wilk test results). Therefore, the data were subjected to one-way ANOVA and post hoc Bonferroni’s tests. The data were demonstrated by mean ± standard deviation (SD). The presence of significance was considered when the *p* value was equal to or less than 0.05. Data were analyzed using GraphPad Prism software (version 9.3.1, San Diego, CA, USA).

## Results

### Neurobehavioral tests

**Fig. 2:**
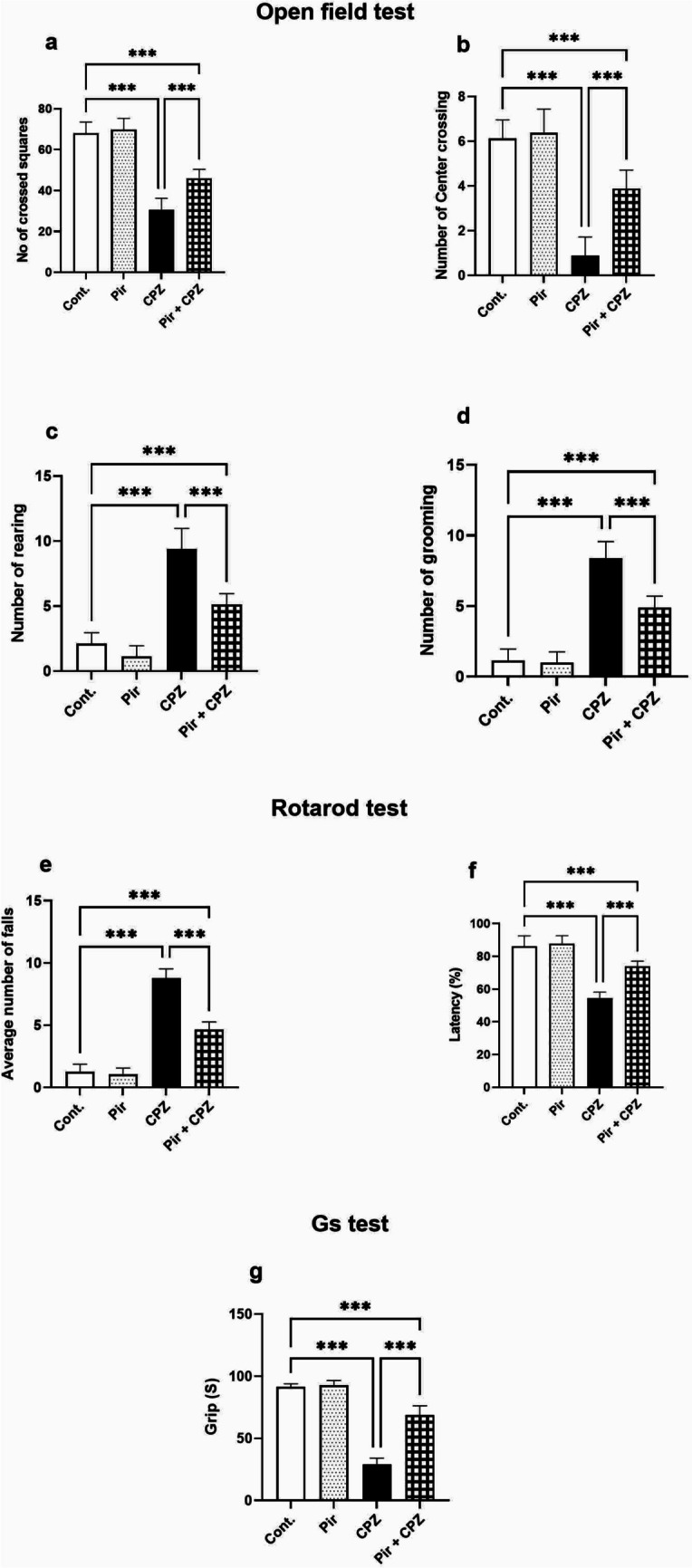
Pir treatment improved neurobehavioral functions induced by CPZ. Pir treatment increased the number of crossed squares (**a**) and center crossing (**b**) while reducing rearing (**c**) and grooming (**d**). Moreover, Pir treatment reduced the average number of falls (**e**) and increased latency % (**f**) in the rotarod test. Pir treatment enhanced motor ability by increasing grip force (**g**). Results were analyzed via ANOVA followed by Bonferroni’s test. Bars represent mean ± SD (*n* = 10). ***P < 0.001. Cont., control; CPZ, cuprizone; GS, grip strength; Pir, pirfenidone

Regarding the open field test, the number of crossed squares and center crossing was non-significant (*P* > 0.05) between the Pir group and the control mice (69.75 ± 5.55 and 6.38 ± 1.06, respectively, vs. 68.00 ± 5.53 and 6.14 ± 0.90, respectively). The number of crossed squares and center crossing decreased significantly (*P* < 0.001) in the CPZ group (30.63 ± 5.60 and 0.88 ± 0.83, respectively) compared with the Cont. group. Meanwhile, they increased significantly (*P* < 0.001) in Pir-treated mice (46.00 ± 4.34 and 3.88 ± 0.83, respectively). However, the number of crossed squares and center crossing in the Pir-treated group were significantly lower than in the control group (*P* < 0.001) (Fig. 2a,b).

Furthermore, rearing and grooming in the Pir group were non-significant (*P* > 0.05) compared with the Cont. group (1.13 ± 0.83 and 1.00 ± 0.76, respectively, vs. 2.13 ± 0.83 and 1.13 ± 0.83, respectively). On the contrary, the CPZ group presented with a significant (*P* < 0.001) increase in rearing and grooming (9.38 ± 1.60 and 8.38 ± 1.19, respectively) compared with the Cont. group. Meanwhile, rearing and grooming in the Pir-treated group decreased significantly (5.13 ± 0.83 and 4.88 ± 0.83, respectively, *P* < 0.001) but were still significantly higher than the Cont. group (*P* < 0.001) (Fig. 2c,d).

Rotarod test results unveiled that the average number of falls and latency percentage were non-significant (*P* > 0.05) between the Pir and Cont. groups (1.04 ± 0.51 and 87.79% ± 4.88, respectively, vs. 1.25 ± 0.62 and 86.20% ± 6.37, respectively). The average number of falls increased significantly (*P* < 0.001), while the latency percentage decreased considerably in the CPZ group (8.79 ± 0.75 and 54.40% ± 3.75, respectively) compared with the Cont. group (*P* < 0.001). The average number of falls decreased in the Pir-treated group, while the latency percentage increased (4.66 ± 0.60 and 73.75% ± 3.32, respectively, *P* < 0.001) compared with the CPZ group. However, in comparison with the Cont. group, the average number of falls and latency percentage in the Pir-treated group were not normalized (*P* < 0.001) (Fig. 2e,f).

Additionally, after performing the GS test, the Pir group’s grip force showed no difference (*P* > 0.05) compared with the Cont. group (92.63 ± 3.96 S vs. 91.50 ± 2.45 S). The CPZ group had a significant (*P* < 0.001) reduction in grip force (29.13 ± 4.97 S) compared with the Cont. group. The grip force was enhanced (*P* < 0.001) in the Pir-treated group (68.63 ± 7.50 S) compared with the CPZ group without attaining the levels of the Cont. group (*P* < 0.001) (Fig. 2g).

### EMG and NCV

**Fig. 3:**
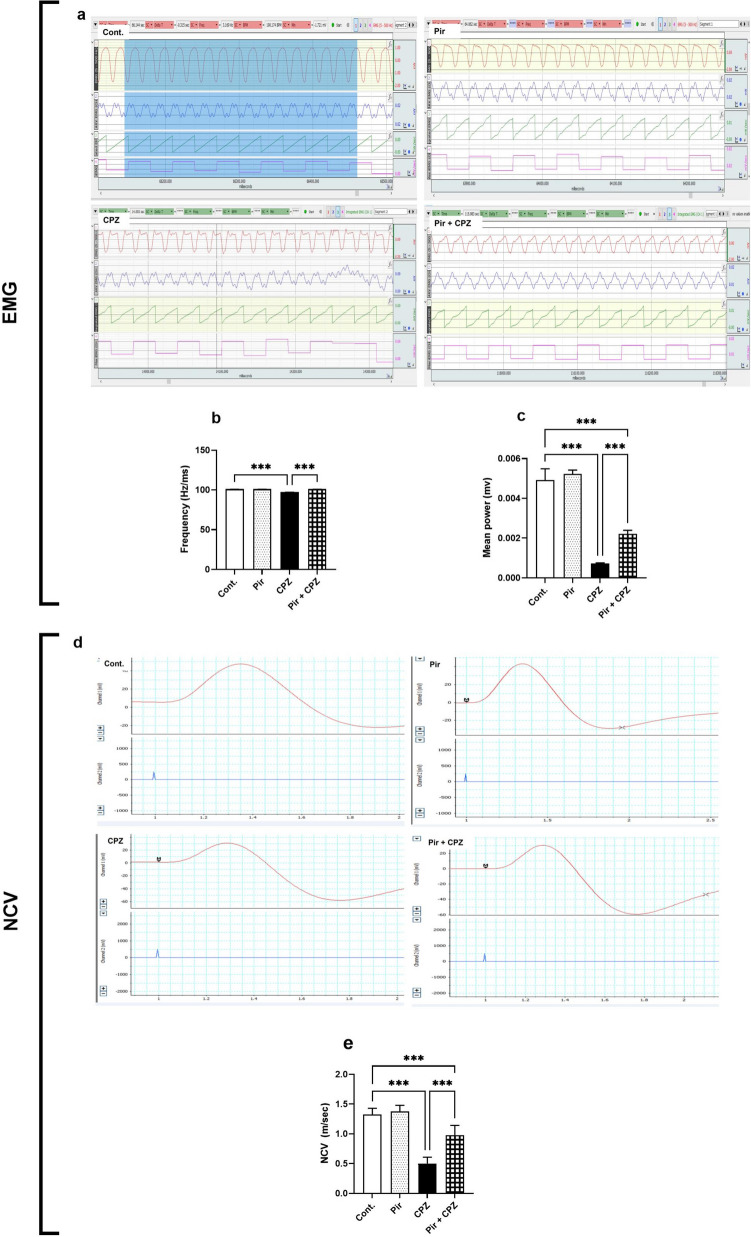
Pir treatment alleviated electrophysiological alterations induced by CPZ. EMG illustrated that Pir treatment improved muscle frequency and power (**a**). Pir increased the score of muscle frequency (**b**) and mean power (**c**). NCV test indicated that mice that received Pir treatment had a marked increase in NCV (**d**) and increased NCV score (**e**). Results were analyzed via ANOVA followed by Bonferroni’s test. Bars represent mean ± SD (*n* = 10). ***P < 0.001. Cont., control; CPZ, cuprizone; EMG, electromyography; NCV, nerve conduction velocity; Pir, pirfenidone

EMG of gastrocnemii muscles revealed a non-significant difference between the Pir and Cont. groups (*P* > 0.05) regarding the mean values of muscle frequency and muscle power (101.12 ± 0.05 Hz and 0.005 ± 0.00 mv, respectively, vs. 101.12 ± 0.06 Hz and 0.005 ± 0.00 mv, respectively). On the contrary, EMG in the CPZ group demonstrated significantly reduced frequency and mean power of the gastrocnemii muscles (97.25 ± 0.15 Hz and 0.001 ± 0.00 mv, respectively, *P* < 0.001), and that was improved (*P* < 0.001) by Pir treatment. In response to Pir therapy (101.10 ± 0.05 Hz and 0.002 ± 0.00 mv, respectively), frequency returned to normal (*P* > 0.05). However, muscle power could not attain the Cont. group levels (*P* < 0.001) (Fig. 3a–c, respectively).

Assessment of NCV of sciatic nerve showed a non-significant difference (*P* > 0.05) between the Pir and Cont. groups (1.38 ± 0.10 m/s vs. 1.33 ± 0.10 m/s, respectively). However, NCV in the CPZ group (0.50 ± 0.11 m/s) decreased significantly (*P* < 0.001) compared with the Cont. group. Pir treatment increased (*P* < 0.001) the NCV (0.98 ± 0.17 m/s) compared with the CPZ group. The response to Pit treatment was not enough to increase NCV as in the Cont. group (*P* < 0.001) (Fig. 3d,e).

### Oxidative stress and inflammatory markers

**Fig. 4:**
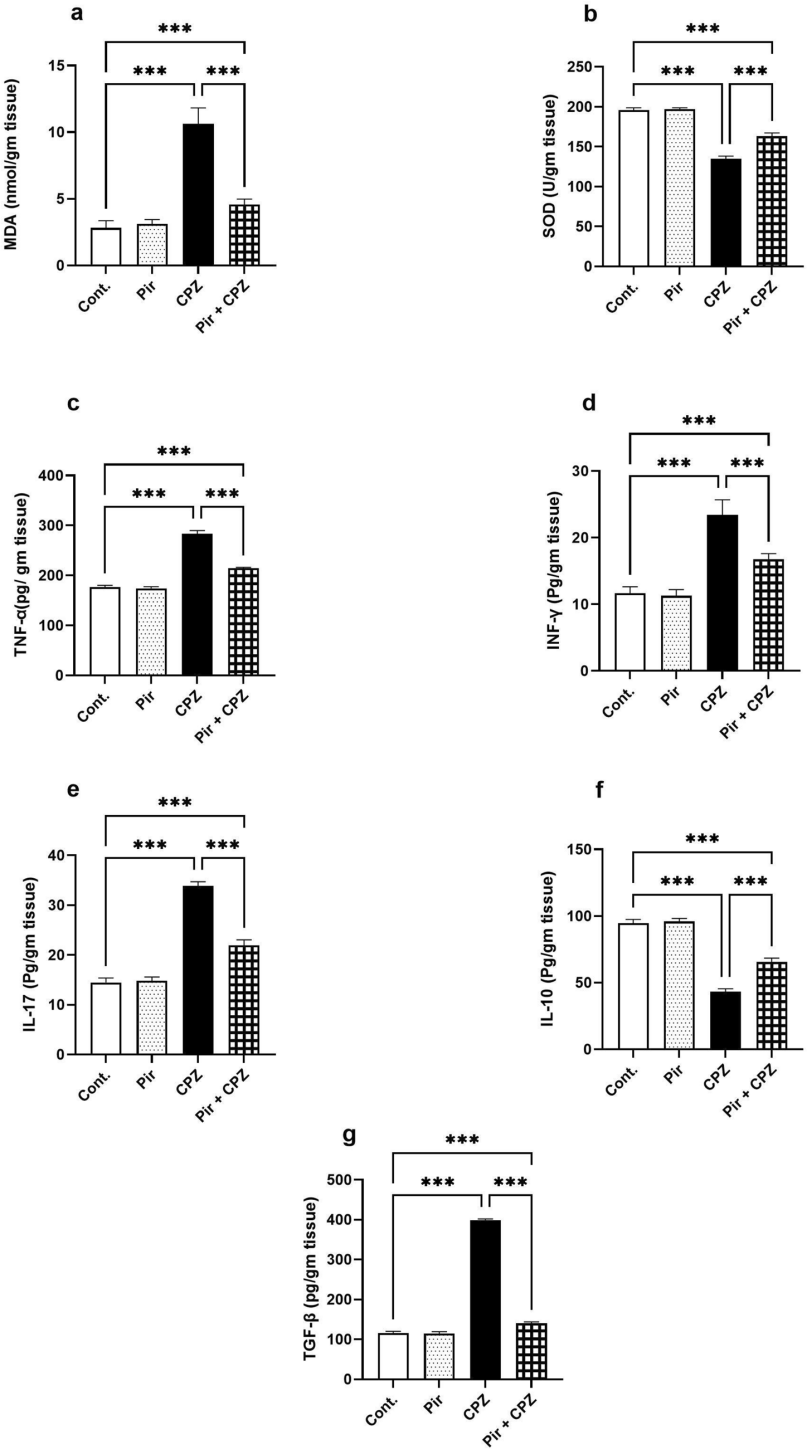
Pir treatment alleviated oxidative stress, inflammation, and fibrosis induced by CPZ. Pir treatment reduced brain MDA (**a**) and increased SOD levels (**b**). Pir treatment had an anti-inflammatory effect as it reduced brain TNF-α (**c**), INF-γ (**d**), and IL-17 (**e**) and increased the anti-inflammatory cytokines IL-10 (**f**). Furthermore, Pir therapy reduced the fibrotic marker TGF- β1 (***g***). Results were analyzed via ANOVA followed by Bonferroni’s test. Bars represent mean ± SD (*n* = 10). ***P < 0.001. Cont., control; CPZ, cuprizone; IL-10, interleukin 10; IL-17, interleukin 17; INF-γ, interferon-γ; MDA, malondialdehyde; Pir, pirfenidone; SOD, superoxide dismutase; TGF-β1, transforming growth factor-β1; TNF-α, tumor necrosis factor-α

Brain lipid peroxidation was evidenced by measuring the brain MDA level. There was a non-significant change (*P* > 0.05) between the Pir and the Cont. groups regarding the brain MDA (3.10 ± 0.34 nmol/gm.tissue vs. 2.81 ± 0.55 nmol/gm.tissue). However, the brain MDA level was elevated in the CPZ group (10.61 ± 1.21 nmol/gm.tissue) compared with the Cont. group. The Pir-treated group showed reduced MDA level (4.56 ± 0.40 nmol/gm.tissue) compared with the CPZ group (P < 0.001). However, the MDA level in the Pir-treated group was still higher than the Cont. group (*P* < 0.001) (Fig. 4a).

The antioxidant enzyme, SOD, showed a non-significant (*P* > 0.05) difference in the Pir group compared with the Cont. group (197.08 ± 1.53 U/gm.tissue vs. 195.81 ± 2.79 U/gm.tissue). On the contrary, SOD levels decreased in the CPZ group compared with the Cont. group (134.65 ± 3.49 U/gm.tissue, *P* < 0.001). The Pir-treated group exhibited a higher SOD level (163.04 ± 4.11 U/gm.tissue, *P* < 0.001) compared with the CPZ group. Notably, the SOD level was considerably lower when compared with the Cont. group (*P* < 0.001) (Fig. 4b).

The difference in the levels of proinflammatory cytokines, TNF-α, INF-γ, and IL-17, was non-significant (*P* > 0.05) in the Pir group compared with the Cont. group (174.23 ± 3.18 pg/gm.tissue, 11.29 ± 0.92 pg/gm.tissue, and 14.80 ± 0.79 pg/gm.tissue, respectively, vs. 176.36 ± 3.73 pg/gm.tissue, 11.66 ± 0.98 pg/gm.tissue, and 14.48 ± 0.91 pg/gm.tissue, respectively). The brain TNF-α, INF-γ and IL-17 levels were elevated in the CPZ group compared with the Cont. group (283.36 ± 6.59 pg/gm.tissue, 23.44 ± 2.24 pg/gm.tissue, and 33.85 ± 0.88 pg/gm.tissue, respectively, *P* < 0.001), whereas treating mice with Pir reversed that elevation (214.61 ± 1.69 pg/gm.tissue, 16.74 ± 0.88 pg/gm.tissue, and 21.90 ± 1.14 pg/gm.tissue, respectively, *P* < 0.001). However, the Pir-treated group’s cytokines levels mentioned above were not normalized (*P* < 0.001) compared with the Cont. group (Fig. 4c–e, respectively).

Regarding the anti-inflammatory cytokine, IL-10, there was a non-significant change (*P* > 0.05) between the Pir and the Cont. groups (95.91 ± 2.28 pg/gm.tissue vs. 94.58 ± 2.89 pg/gm.tissue, respectively). On the other hand, IL-10 showed a pronounced reduction in the brains of the CPZ-treated mice compared with the Cont. mice (43.25 ± 2.23 pg/gm.tissue, *P* < 0.001). The decline in the IL-10 level was improved in response to treatment with Pir (65.43 ± 3.07 pg/gm.tissue, *P* < 0.001). However, the levels of IL-10 in the Pir-treated group were still substantially lower (*P* < 0.001) compared with the Cont. group (Fig. 4f).

TGF-β1 brain levels were almost the same in the Pir-treated and the Cont. groups (114.5 ± 4.95 pg/gm.tissue vs. 115.5 ± 4.54 pg/gm.tissue, *P* > 0.05). Fibrosis was confirmed by the profound increase in TGF-β1 levels in the CPZ group (398.89 ± 3.09 pg/gm.tissue, *P* < 0.001) relative to the Cont. group. Pir treatment (140.1 ± 8.81 pg/gm.tissue) significantly (*P* < 0.001) mitigated TGF-β1 compared with the CPZ-treated mice. Unfortunately, TGF-β1 levels were not restored by Pir therapy as compared with normal mice (*P* < 0.001) (Fig. 4g).

### Brain expression of NF-κB, SIRT1, NGF, and neuregulin-1 expression using RT-PCR

**Fig. 5:**
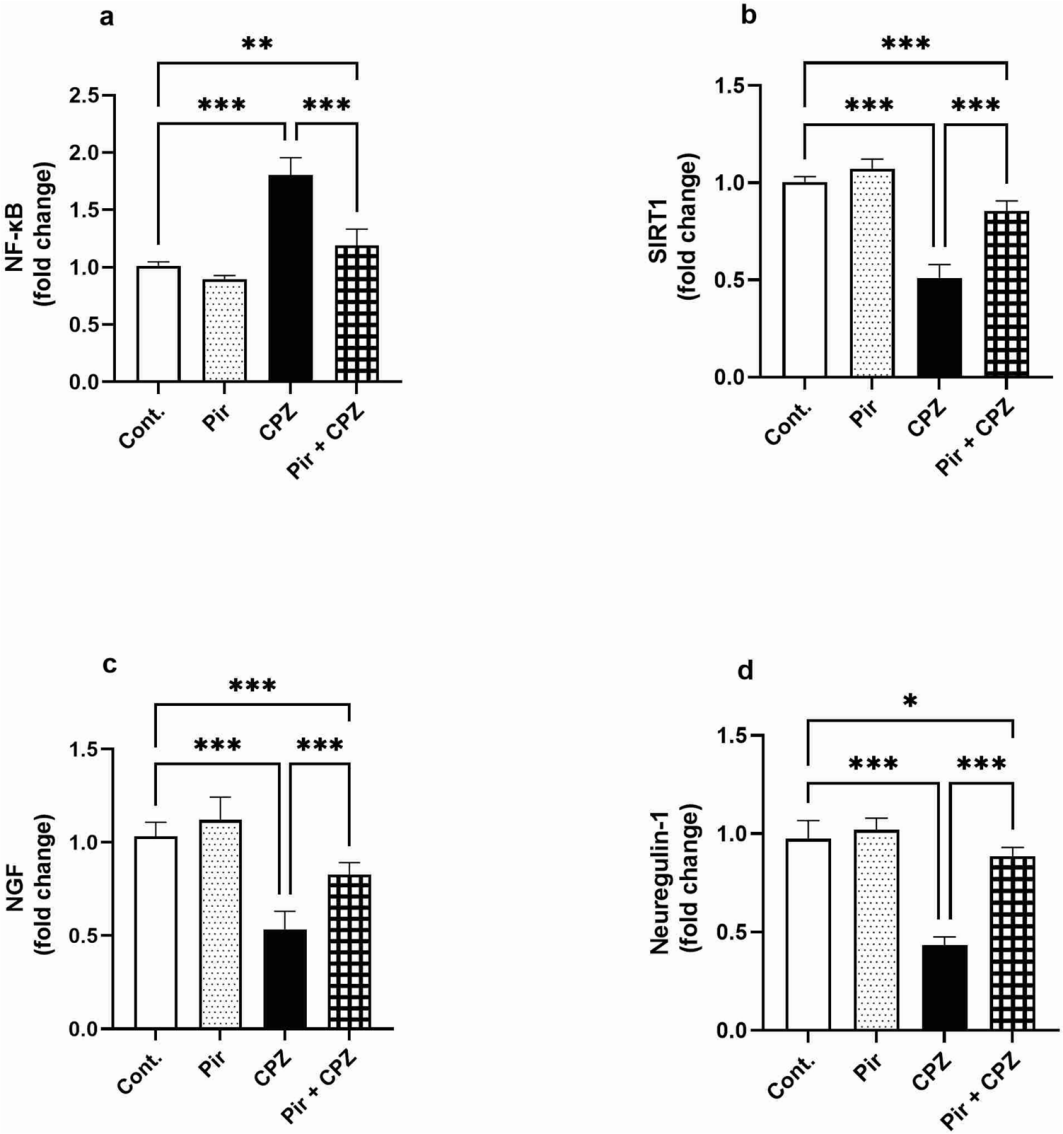
Pir treatment modulated CPZ-induced impairment of NF- κB, SIRT1, NGF, and neuregulin-1 expression. Pir treatment lowered NF-κB expression (**a**). Pir treatment increased SIRT1 (**b**), NGF (**c**), and neuregulin-1 (**d**). Results were analyzed via ANOVA followed by Bonferroni’s test. Bars represent mean ± SD (*n* = 10). *P < 0.05, **P < 0.01, and ***P < 0.001. Cont., control; CPZ, cuprizone; NGF, nerve growth factor; NF-κB, nuclear factor kappa B; Pir, pirfenidone; SIRT1, sirtuin 1

The expression of NF-ҝB, SIRT1, NGF, and neuregulin-1 in the mice’s brains was non-significant (*P* > 0.05) in the Pir group compared with the Cont. group (0.90 ± 0.04, 1.07 ± 0.05, 1.10 ± 0.11, and 1.02 ± 0.06, respectively, vs. 1.01 ± 0.03, 1.00 ± 0.03, 1.02 ± 0.08, and 0.97 ± 0.09, respectively). The CPZ group mice had a significant (*P* < 0.001) increase in NF-κB expression compared with the Cont. group mice (1.81 ± 0.15, *P* < 0.001) which was reversed by Pir treatment (1.19 ± 0.17). Pir-treated mice had a significantly elevated NF-κB expression level compared with the control mice (*P* < 0.01). In addition, the CPZ group exhibited significantly lower SIRT1, NGF, and neuregulin-1 expression (0.51 ± 0.07, 0.49 ± 0.04, and 0.43 ± 0.04, respectively, *P* < 0.001) than the Cont. group. In response to Pir treatment, these gene expressions were elevated (0.86 ± 0.05, 0.81 ± 0.07, and 0.89 ± 0.05, respectively, *P* < 0.001) compared with the CPZ group. However, their levels were still lower than the Cont. group (*P* < 0.001, *P* < 0.001, and *P* < 0.05, respectively) (Fig. 5a–d, respectively).

### Histopathological and immunohistochemical results of mice corpus callosum

**Fig. 6:**
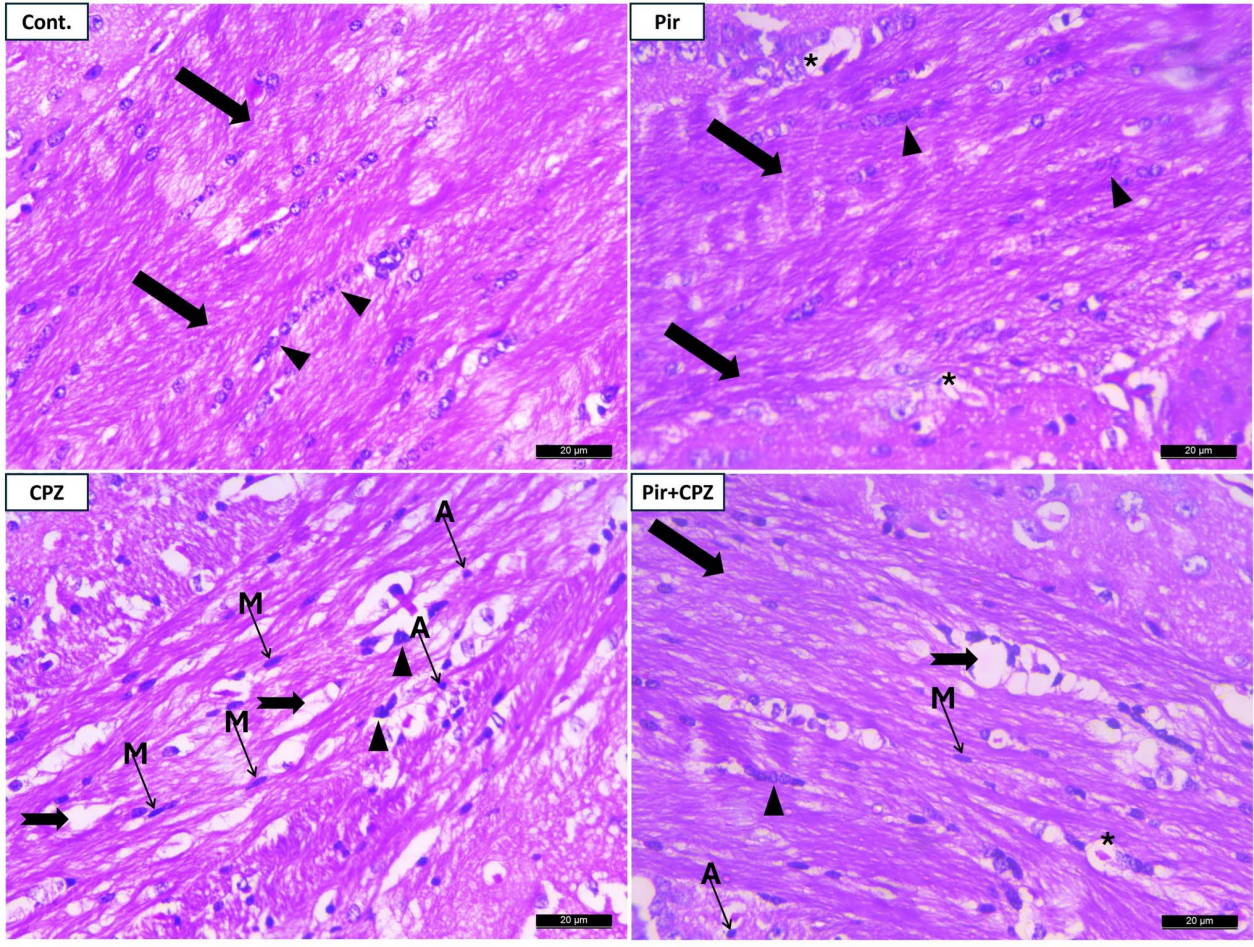
Representative H&E staining of Cont. and Pir mice groups shows densely packed, myelinated, and parallel-arranged fibers (arrows) and oligodendrocytes nuclei (arrowheads) arranged in longitudinal rows parallel to the nerve fibers that they myelinate. Well-defined, rounded, darkly stained nuclei are exhibited. Tiny capillaries (stars) can be detected in the group. The CPZ group shows areas with vacuolation, demyelination, axonal damage (notched arrows), and distorted oligodendroglia (arrowhead). Microglial (M) and astroglial cells (A) infiltrates could be detected. The Pir + CPZ group reveals myelinated fibers (arrows), oligodendrocytes nuclei (arrowheads), and tiny capillaries (star). However, demyelination with axonal damage (notched arrows), few microglia, and astrocytes are still detected (×400, scale bar = 20 μm)

Light microscopic examination of the corpus callosum of the Cont. and Pir groups displayed a densely packed, myelinated, parallel arrangement of myelinated nerve fibers. A longitudinal row of oligodendrocytes appears parallel to the myelin nerve fibers, showing rounded, well-defined, and darkly stained nuclei (Fig. 6).

Multiple areas of partially demyelinated nerve fibers were observed in the CPZ-treated group, with axonal disruptions and splitting and partial loss of oligodendrocytes nuclei, which were dispersed and showed faint staining. Others appeared circumscribed and darkly stained. Few oligodendrocyte nuclei were seen oriented parallel to the nerve fibers. Infiltration with astrocytes and microglia could be detected (Fig. 6).

Most nerve fibers in the Pir-treated group exhibited fair myelination and appeared well-packed and arranged. In some specimens, axonal disruption and fragmentation were evident, and the oligodendrocyte nuclei appeared disorganized. A few oligodendrocytes were arranged in linear rows parallel to the nerve fibers. Most nuclei were rounded and darkly stained, but a few were flattened, small, and faintly stained (Fig. 6).

**Fig. 7:**
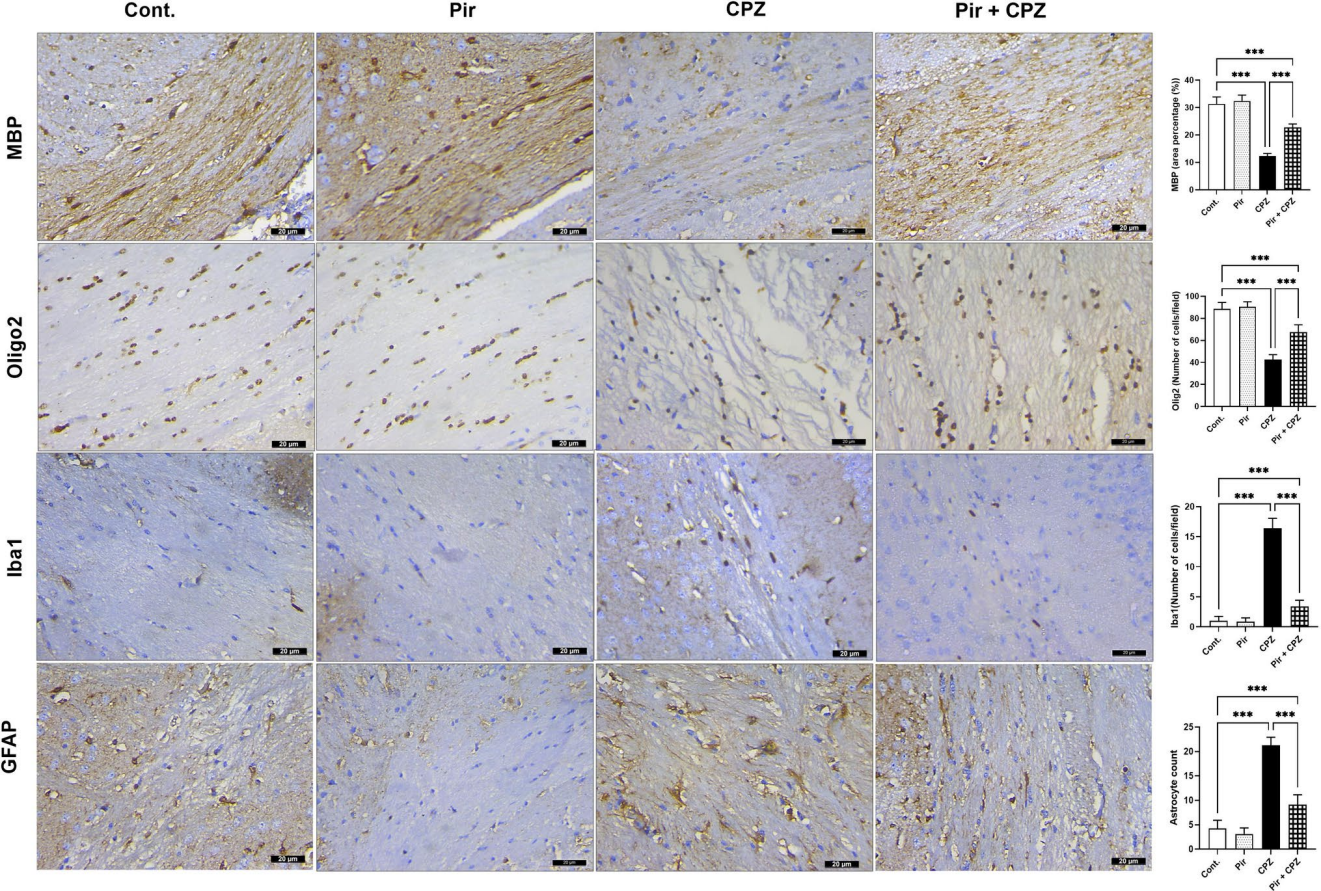
Expression of MBP, Olig2, Iba1, and GFAP in the corpus callosum of the Cont., Pir, CPZ, and Pir + CPZ groups. Scale bar = 20 μm. Results were analyzed via ANOVA followed by Bonferroni’s test. Bars represent mean ± SD (*n* = 10). ***P < 0.001. Cont., control; CPZ, cuprizone; GFAP, glial fibrillary acidic protein antibody; Iba1, anti-ionized calcium-binding adapter molecule 1; MBP, myelin basic protein; Oligo2, oligodendrocyte transcription factor 2; Pir, pirfenidone

MBP of the mice’s corpus callosum and Oligo2 were of no difference in the Pir group compared with the Cont. mice (32.43 ± 2.15 and 90.63 ± 4.47 vs. 31.34 ± 2.57 and 88.75 ± 5.78, respectively, *P* > 0.05). CPZ markedly reduced the expression of MBP and Oligo2 in the brains of mice (12.33 ± 0.99 and 42.63 ± 4.53, respectively, *P* < 0.001) compared with the Cont. mice. Meanwhile, Pir therapy managed to elevate the expression of these markers (22.83 ± 1.23 and 67.38 ± 6.84, respectively, *P* < 0.001) compared with the CPZ mice. The expression of Olig2 and MBP in the Pir-treated group was considerably lower than in the Cont. group (*P* < 0.001) (Fig. 7).

Moreover, The Iba1 counts and GFAP expression witnessed no difference in the Pir group compared with the Cont. group (0.88 ± 0.64 vs. 1.00 ± 0.76 and 3.13 ± 1.25 vs. 4.25 ± 1.67, respectively, *P* > 0.05). CPZ caused a marked increase in Iba1 expression (16.38 ± 1.69) and GFAP count (21.25 ± 1.67) compared with the normal mice (*P* < 0.001). Conversely, Pir treatment lowered these parameters (3.38 ± 1.06 and 9.13 ± 2.03, respectively, *P* < 0.001) compared with the CPZ-treated mice. Unfortunately, Pir could not reach the optimum values of the Cont. group (*P* < 0.001) (Fig. 7).

### Histopathologic and immunohistochemical evaluation of sciatic nerve sections

**Fig. 8:**
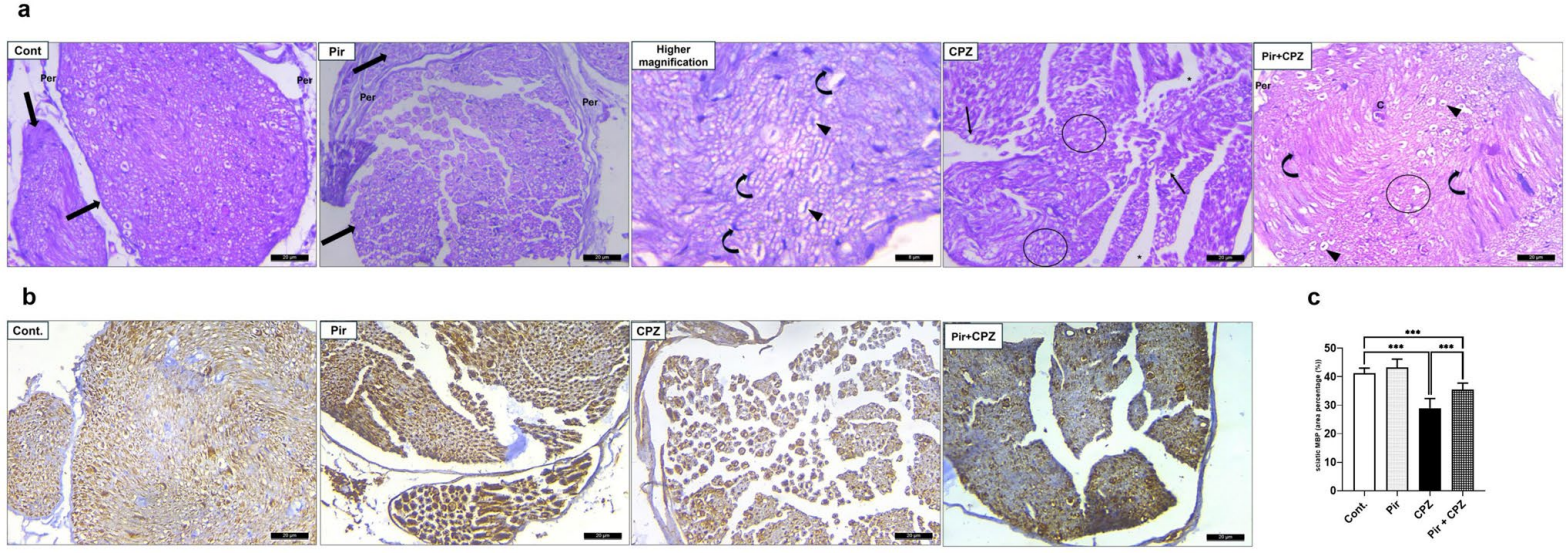
**a** Representative H&E staining of Cont. and Pir groups show a transverse section of the sciatic nerve formed by the nerve fascicle (arrows) surrounded by the perineurium (per). Higher magnification reveals myelinated nerve fibers formed of axons surrounded by unstained areas of dissolved myelin (arrowheads) and nuclei of Schwann cells (curved arrows). The CPZ group shows a distortion of the normal architecture of a sciatic nerve fascicle. There is a wide separation of nerve fibers from each other (stars). Some are disorganized (circles), and others have discontinuous neurilemmal sheaths (arrows). The Pir + CPZ group reveals the nearly normal architecture of the sciatic nerve fascicle surrounded by perineurium (per). Nerve fibers appear as axons surrounded by unstained myelin (arrowheads) and nuclei of Schwann cells (curved arrows). However, focal areas of mildly disorganized nerve fibers (circles) are still detected. **b** Expression of MBP in the sciatic nerve of the Cont., Pir, CPZ, and Pir + CPZ groups. Scale bar = 20 μm. **c** Mean score of MBP expression in sciatic nerves. Results were analyzed via ANOVA followed by Bonferroni’s test. Bars represent mean ± SD (*n* = 10). ***P < 0.001. Cont., control; CPZ, cuprizone; MBP, myelin basic protein; Pir, pirfenidone

H&E-stained sections of Cont. and Pir groups revealed bundles of nerve fibers, each wrapped by a thin layer of connective tissue called perineurium. The myelinated nerve fiber is composed of unstaining dissolved myelin surrounding axoplasm. Nuclei of Schwann cells appeared between nerve fibers, as well as unmyelinated nerve fibers. In the CPZ-treated group, the sciatic nerve displayed loss of regular architecture and separation of nerve fibers from each other and overlying perineurium. Pir + CPZ-treated mice displayed normal nerve fascicles containing Schwann cell nuclei and myelinated nerve fibers. The nerve fibers, however, remained widely separated (Fig. 8a).

MBP expression of sciatic nerves was of no difference in the Pir group compared with the Cont. mice (43.26 ± 2.92 vs. 41.15 ± 1.87, *P* > 0.05). CPZ markedly reduced the MBP expression in mice’s sciatic nerve (28.95 ± 3.38, *P* < 0.001) compared with the Cont. mice. Meanwhile, Pir therapy managed to increase the expression of this marker (35.45 ± 2.30, *P* < 0.001) compared with the CPZ mice. The expression of MBP in the Pir-treated group was considerably lower than in the Cont. group (*P* < 0.001) (Fig. 8b,c).

## Discussion

There are currently no authorized MS medications that may reverse or stabilize the neurological impairments linked to this illness. Recognizing illness signs and streamlining clinical evaluation are more accessible by understanding MS processes. CPZ is widely utilized to induce demyelination and oligodendrocyte cell loss like MS (Zhen et al. 2017).

The present study showed a significant increase in the levels of proinflammatory cytokines (TNF-α, INF-γ, IL-17, and TGF-β) and NF-kB expression in the brain, accompanied by a significant decrease in the anti-inflammatory cytokine level of IL-10 in the CPZ group in comparison with the control group. CPZ causes chelation of copper, resulting in an alteration in the metabolic process of oligodendrocytes; cells are responsible for the axonal myelination in the CNS and induce selective loss of oligodendrocytes (Polyák et al. 2023). Myelin damage, a hallmark of MS, triggers an inflammatory response mediated by various immune cells and cytokines. Resident immune cells such as microglia and astrocytes recognize myelin debris and other damage-associated molecules (Kent and Miron 2024). These cells activate and release proinflammatory cytokines, such as TNF-α and INF-γ. Proinflammatory cytokines attract and activate peripheral immune cells, infiltrating the lesion site and contributing to myelin and axonal damage by releasing cytotoxic molecules and proteolytic enzymes. This sustained inflammation perpetuates a vicious cycle of myelin destruction, axonal injury, and neurodegeneration (Varas and Ortiz 2019).

TGF-β1 and its receptors are found in the CNS of MS patients. TGF-β1 is a crucial element for the differentiation of TH17, which secretes IL-17, indicating a pathogenic involvement of TGF-β1 in MS (Mirshafiey and Mohsenzadegan 2009). Interleukin-17 plays a vital role in MS etiology. Upon binding to its receptor, IL-17 activates several signaling pathways, including NF-κB (Safwat et al. 2023). NF-κB activation in macrophages and microglia promotes proinflammatory cytokine secretion. Astrocytes exhibit upregulated NF-κB in MS lesions, suggesting a detrimental role, although blocking NF-κB in astrocytes has shown therapeutic potential (Zhou et al. 2020). The protective role of IL-10 is attributed to modulating the immune response, promoting tissue repair, and supporting re-myelination processes (Wang et al. 2018). Our histopathological and immunohistochemical results support these potential mechanisms that revealed demyelinated nerve fibers of the corpus callosum with decreased oligodendrocytes count. The number of microglia and astrocytes significantly increased, as revealed in the previous studies (Zhen et al. 2017).

Pir treatment ameliorated neuroinflammation in our results. These findings align with previous studies, which concluded that Pir exerts its anti-inflammatory effects by suppressing the NF-κB pathway (Zhang et al. 2023). Additionally, Antar et al. indicated that Pir reduces inflammation by inhibiting the production of TNF-α, IL-1β, and IL-6 (Antar et al. 2022). Ruwanpura et al. revealed that Pir upregulated IL-10 by downregulating inflammatory pathways, targeting inflammasome pathways, and suppressing TGF-β1, highlighting its potential as an anti-inflammatory agent (Ruwanpura et al. 2020). Experimental evidence has demonstrated that Pir is a collagen and TGF-β production blocker and an activator of MMPs, thus modulating the fibrogenic pathway (Macías-Barragán et al. 2010).

Neuroinflammation induces oxidative stress via the overproduction of ROS by activated microglia and astrocytes. The microglia stimulate NADPH oxidase activity, which generates superoxide and intracellular ROS. Oxidative stress, in turn, induces the activation of the NF-κB pathway, which amplifies the inflammatory state leading to neurodegeneration. Oligodendroglia are susceptible to death due to oxidative stress. Moreover, the proinflammatory and pro-oxidative markers in the CSF resulted in synaptic hyperexcitability and consequent glutamate-dependent neurotoxicity (Lamloum et al. 2023). SIRT1 accelerates the detoxification of ROS by upregulating cellular antioxidant enzymes, including SOD, catalase, and glutathione peroxidase. It also stimulates mitochondrial biogenesis. Toxic chemicals directly inhibit SIRT1 expression (Ren et al. 2019). Downregulation of SIRT1 also activates NF-κB and neuroinflammation (Kauppinen et al. 2013). The aforementioned data were in line with our findings as the CPZ group demonstrated significantly elevated MDA levels, associated with a significant reduction of SOD level and SIRT1 expression in the brain. MDA is a widely utilized biomarker that assesses the extent of lipid peroxidation and oxidative stress (Nicola et al. 2024).

Notably, the Pir + CPZ group demonstrated significant amelioration of oxidative stress state compared with the CPZ group. Pir’s ability to re-establish the oxidative balance could be attributed to its anti-inflammatory properties (Fois et al. 2020). This finding is supported by Kong and Deng, who mentioned that Pir reduces oxidative stress by restoring antioxidant enzyme activities such as SOD and glutathione peroxidase through SIRT1 upregulation (Kong and Deng 2022). Pir’s ability to upregulate SIRT1 occurs via AMPK activation (Sandoval-Rodriguez et al. 2020). Preclinical and clinical findings indicated that SIRT1 upregulation reduces autoimmunity, neurodegeneration, and neuroexcitation. SIRT1 has been shown to promote axonal integrity and synaptic plasticity by regulating the expression of cytoskeletal proteins, axonal transport, and neurotrophic factors (Sharma et al. 2021a). In oligodendrocytes, SIRT1 has been implicated in modulating the expression of myelin-related genes. By promoting neuronal survival, axonal maintenance, and oligodendrocyte function, SIRT1 may contribute to the preservation of myelin integrity and facilitate remyelination processes in demyelinating disorders (Li et al. 2020a). Pir’s multifaceted actions on oxidative stress pathways make it a promising agent for mitigating oxidative damage in various conditions. The histopathological findings go hand in hand with the biochemical findings as there was significant attenuation in the demyelination process, MBP expression was upregulated, and the number of oligodendrocytes increased. The microglia and astrocytes decreased in number, as revealed by Li et al. (2020a).

Furthermore, we investigated the effects of CPZ-induced demyelination and Pir treatment on the expression of neurotrophic genes, NGF and neuregulin-1, which are crucial for promoting neuronal survival and remyelination processes (Dermitzakis et al. 2022). The results of the present study demonstrated that CPZ resulted in a significant downregulation of NGF and neuregulin-1 expression in the brain. The reduction in the expression of these neuroprotective and pro-remyelinating factors can contribute to neuronal injury, axonal degeneration, and impaired remyelination observed in MS (Li et al. 2020b).

Numerous researchers explained the neuroprotective effects of NGF and neuregulin-1 genes. Sang et al. stated that NGF binds to its specific receptors, such as TrkA, on neurons and activates neurite outgrowth and synaptic plasticity. These signaling cascades involve the activation of kinases such as PI3K/Akt and MAPK, ultimately leading to the transcription of pro-survival genes and the inhibition of apoptotic pathways (Sang et al. 2018). Moreover, NGF has been shown to enhance the expression of cytoskeletal proteins and promote axonal transport, thereby supporting axonal integrity and maintaining efficient communication between neurons and their targets. In demyelinating disorders, NGF may help protect neurons from ongoing inflammation and demyelination (Brandi et al. 2021).

Neuregulin-1 is a growth factor that neurons produce and acts on oligodendrocytes to enhance myelination. Neuregulin-1 binds to its receptors, ErbB3 and ErbB4, on oligodendrocyte precursor cells (OPCs), activating their differentiation into mature, myelinating oligodendrocytes (Ding et al. 2021). Additionally, neuregulin-1 has been shown to enhance the survival of mature oligodendrocytes, thereby supporting the maintenance of existing myelin sheaths. In demyelinating disorders, neuregulin-1 may play a crucial role in promoting remyelination by stimulating the recruitment and differentiation of OPCs to replace damaged myelin (Zhou and Zhang 2023). Kataria et al. demonstrated that reduced plasma levels of neuregulin-1 are positively linked to progression to MS, and restoring its levels delays disease onset and mitigates symptoms (Kataria et al. 2021).

Conversely, Pir treatment reinstated the expression of NGF and neuregulin-1 in our findings. Pir may restore NGF expression by attenuating NF-κB activity, preventing its downregulation induced by inflammation (Silva-Gomez et al. 2021). Furthermore, Pir’s antioxidant effects help protect neurons from oxidative damage, which can enhance the responsiveness of neuregulin-1 signaling pathways (Graziani et al. 2021). Overall, Pir’s multifaceted effects on these molecular pathways collectively contribute to its potential in neuroprotection and remyelination in MS.

In the present study, we assessed the behavioral changes and motor deficits associated with MS by employing various neurobehavioral tests. The open field test is a widely used behavioral assay to evaluate rodents’ locomotor activity and anxiety-like behavior (Kraeuter et al. 2019). The present study demonstrated that CPZ caused a significant reduction in the number of crossed squares, center crossing, and increased rearing and grooming, indicating decreased locomotor activity and increased anxiety-like behavior. Reduced locomotor activity and exploratory behavior could be attributed to the disruption of neuronal communication and impaired motor function resulting from myelin damage (Mitra et al. 2020). The observed neurobehavioral changes could be linked to CPZ-induced selective loss of oligodendrocytes in MS. Observation of MS patients has indicated apoptosis of oligodendrocytes (Polyák et al. 2023) in addition to the apparent neuroinflammation and oxidative stress, shared in neurodegeneration, as confirmed by our results.

Interestingly, Pir treatment ameliorated neurobehavioral deficits and improved motor function, suggesting its potential to improve locomotor activity and reduce anxiety. This improvement suggests that Pir may exert neuroprotective and remyelinating effects, thereby preserving neuronal function. Pir’s ability to reduce neuroinflammation, oxidative stress, and gliosis contributes to its neuroprotective effects (Antar et al. 2022).

The results of the rotarod test revealed that CPZ administration increased the frequency of falls and decreased the latency percentage, indicating compromised motor coordination and balance. Additionally, the grip strength test showed a notable decline in muscular strength. However, Pir treatment partially reversed the motor disabilities, aligning with other studies that found that Pir enhances functional recovery following compression spinal cord injury (Han et al. 2020). Pir may improve muscular coordination and function through its antifibrotic effects. In conditions like Duchenne muscular dystrophy (DMD), where fibrotic tissue replaces muscle fibers, Pir has shown promise in reducing fibrosis, cell proliferation, migration, and collagen secretion in muscle-derived fibroblasts (Rawls et al. 2023). Additionally, Castro-Torres et al. reported Pir’s ability to attenuate microglial reactivity and reduce inducible nitric oxide synthase expression in the hippocampus after excitotoxicity indicates its potential to modulate inflammation and oxidative stress, which are crucial factors affecting muscle coordination and function (Castro-Torres et al. 2015).

EMG and NCV measurements provide insights into muscle and nerve function, respectively. The EMG and NCV results demonstrated that CPZ leads to impairments in nerve and muscle functions. Demyelination can significantly disrupt nerve conduction and induce muscle weakness through several mechanisms, including impaired saltatory conduction, which slows or blocks nerve impulse transmission; altered properties of muscle fibers, such as changes in fiber type composition and metabolic characteristics that weaken the muscle; and disrupted neuromuscular transmission by affecting the structure and function of the neuromuscular junction. These demyelination-induced disruptions in axonal conduction, muscle fiber integrity, and neuromuscular transmission contribute to the motor impairments and muscle dysfunction observed in MS (Liu et al. 2020).

The histopathological findings of the sciatic nerve coincide with those of the NCV, as there was a loss of regular architecture, separation of its nerve fibers from each other and the overlying perineurium, and decreased expression of MBP. The same result was reported by other studies (Safwat et al. 2023).

The results illustrated that Pir treatment improved muscle and nerve function, restored nerve structure, and increased MBP expression. This was likely mediated by its multifaceted mechanisms, including anti-inflammatory, antioxidant, and antifibrotic effects **(**Antar et al. 2022**)**, and regulation of gene expression such as NF-κB and SIRT1 related to inflammation, neuronal survival, and remyelination processes (Manawy et al. 2024).

In summary, the findings of the current study highlight Pir’s potential to exert neuroprotective, pro-remyelinating, anti-inflammatory, antioxidant, and antifibrotic effects by modulating the expression of principal genes involved in neuronal survival, axonal integrity, remyelination processes, and inflammatory pathways.

## Conclusion

Our findings provide credibility to using CPZ in mice models for MS-related investigations. Pir improves behavioral abnormalities associated with anxiety, motor coordination, muscle strength, and locomotor activity in the CPZ-induced demyelination model. Moreover, mitigating the electrophysiological changes brought on by demyelination maintains the function of muscles and nerves. One possible explanation could be its capacity to reduce oxidative stress, fibrosis, and neuroinflammation. These effects may be mediated by altering the neurotrophic genes SIRT1, NF-κB, NGF, and neuregulin-1 pathways. Based on our findings, we believe that Pir is a promising treatment for MS.

## Data Availability

No datasets were generated or analysed during the current study.
